# Phase Ib study evaluating safety and clinical activity of the anti-HER3 antibody lumretuzumab combined with the anti-HER2 antibody pertuzumab and paclitaxel in HER3-positive, HER2-low metastatic breast cancer

**DOI:** 10.1007/s10637-018-0562-4

**Published:** 2018-01-19

**Authors:** Andreas Schneeweiss, Tjoung-Won Park-Simon, Joan Albanell, Ulrik Lassen, Javier Cortés, Veronique Dieras, Marcus May, Christoph Schindler, Frederik Marmé, Juan Miguel Cejalvo, Maria Martinez-Garcia, Iria Gonzalez, Jose Lopez-Martin, Anja Welt, Christelle Levy, Florence Joly, Francesca Michielin, Wolfgang Jacob, Céline Adessi, Annie Moisan, Georgina Meneses-Lorente, Tomas Racek, Ian James, Maurizio Ceppi, Max Hasmann, Martin Weisser, Andrés Cervantes

**Affiliations:** 1 National Center for Tumor Diseases, Heidelberg University Hospital, Heidelberg, Germany; 2 Department of Obstetrics and Gynecology, Division of Gynecological Oncology and Clinical Research Center, Hannover Medical School, Hannover, Germany; 3 Department of Medical Oncology, Hospital del Mar, CIBERONC, Barcelona, Spain; 4 Rigshospitalet, Copenhagen, Denmark; 5 Ramon y Cajal University Hospital, Madrid, Spain; 6 Vall d’Hebron Institute of Oncology, Barcelona, Spain; 7 Department of Medical Oncology, Institute Curie, Paris, France; 8 Department of Medical Oncology, Biomedical Health Research Institute INCLIVA, University of Valencia, Valencia and CIBERONC, Institute of Health Carlos III, Madrid, Spain; 9 Department of Medical Oncology, Hospital Universitario 12 de Octubre, Madrid, Spain; 10 Department of Medical Oncology, West German Cancer Center, University Hospital Essen, Essen, Germany; 11 Departments of Clinical Research Unit and Medical Oncology, Centre François Baclesse, Caen, France; 12 Pharma Research and Early Development (pRED), Roche Innovation Center Basel, Basel, Switzerland; 13 Pharma Research and Early Development (pRED), Roche Innovation Center Munich, Penzberg, Germany; 14 Pharma Research and Early Development (pRED), Roche Innovation Center Welwyn, Welwyn Garden City, UK; 15 A4P Consulting Ltd, Sandwich, UK

**Keywords:** Human epidermal growth factor receptor 3 (HER3), ErbB3, Phase I, Human epidermal growth factor receptor 2 (HER2), Heregulin (HRG), Pertuzumab, Metastatic breast cancer, Biomarker

## Abstract

*Purpose* To investigate the safety and clinical activity of comprehensive human epidermal growth factor receptor (HER) family receptor inhibition using lumretuzumab (anti-HER3) and pertuzumab (anti-HER2) in combination with paclitaxel in patients with metastatic breast cancer (MBC). *Methods* This phase Ib study enrolled 35 MBC patients (first line or higher) with HER3-positive and HER2-low (immunohistochemistry 1+ to 2+ and in-situ hybridization negative) tumors. Patients received lumretuzumab (1000 mg in Cohort 1; 500 mg in Cohorts 2 and 3) plus pertuzumab (840 mg loading dose [LD] followed by 420 mg in Cohorts 1 and 2; 420 mg without LD in Cohort 3) every 3 weeks, plus paclitaxel (80 mg/m^2^ weekly in all cohorts). Patients in Cohort 3 received prophylactic loperamide treatment. *Results* Diarrhea grade 3 was a dose-limiting toxicity of Cohort 1 defining the maximum tolerated dose of lumretuzumab when given in combination with pertuzumab and paclitaxel at 500 mg every three weeks. Grade 3 diarrhea decreased from 50% (Cohort 2) to 30.8% (Cohort 3) with prophylactic loperamide administration and omission of the pertuzumab LD, nonetheless, all patients still experienced diarrhea. In first-line MBC patients, the objective response rate in Cohorts 2 and 3 was 55% and 38.5%, respectively. No relationship between HER2 and HER3 expression or somatic mutations and clinical response was observed. *Conclusions* Combination treatment with lumretuzumab, pertuzumab and paclitaxel was associated with a high incidence of diarrhea. Despite the efforts to alter dosing, the therapeutic window remained too narrow to warrant further clinical development. Trial registration: on ClinicalTrials.gov with the identifier NCT01918254 first registered on 3rd July 2013.

**Electronic supplementary material:**

The online version of this article (10.1007/s10637-018-0562-4) contains supplementary material, which is available to authorized users.

## Background

In human epidermal growth factor receptor 2 (HER2)-overexpressing metastatic or advanced breast cancer (BC), defined as: immunohistochemistry (IHC) 3+ or positive by in situ hybridization (ISH), treatments with HER2-targeting monoclonal antibodies trastuzumab, pertuzumab and the antibody-drug conjugate trastuzumab emtansine (TDM-1) have demonstrated significantly increased response rates and prolongation of progression-free survival (PFS) and overall survival [1–6].

However, the majority of BC tumors do not show overexpression of the HER2 protein or HER2 gene amplification. For these patients, especially for those in need of systemic therapy for metastatic breast cancer (MBC), therapeutic options are limited. Anthracycline- or taxane-based chemotherapy is indicated as initial therapy for patients with hormone receptor (HR)-negative disease and following failure of hormonal therapies in HR-positive disease [7]. Apart from anti-hormonal agents, targeted therapies are currently not established as standard of care for these patients emphasizing the significant need for new agents and/or novel mechanisms of action.

Upregulation of HER3 signaling provides an “escape route” via which tumor cells may overcome the inhibition of individual HER family members or downstream signaling components of the PI3K-AKT-mTOR signaling pathway [8]. Previous reports suggested that increased HER3 expression was associated with decreased survival of patients with BC [9]. Our hypothesis was that in HER3-positive, HER2-low expressing MBC, tumor growth would depend mainly on heterodimerization of HER family receptors in contrast to HER2-overexpressing MBC where HER2 signaling is the major driver of tumor growth. In the absence of a single driver like amplified HER2, a comprehensive inhibition of HER family dimers may be required to inhibit tumor growth. This could be achieved by combining the HER3-binding antibody lumretuzumab with the HER2 dimerization inhibitor pertuzumab, which would block all possible heterodimers among EGFR, HER2 and HER3.

Clinically, both lumretuzumab [10] and pertuzumab [11] given as single agents have demonstrated favorable safety profiles but limited clinical activity in the setting of HER2-non-overexpressing MBC. Additive tumor growth inhibition and tumor regression was observed in subcutaneous BC xenograft models expressing both HER3 and HER2 (HER2-amplified and non-amplified), as well as in estrogen receptor-positive and triple-negative BC models [12, 13]. Preclinical models demonstrated superior antitumor activity when lumretuzumab was combined with pertuzumab as compared to the combination with trastuzumab or TDM-1, as well as superior antitumor activity compared to any of the mentioned single agents (data not shown).

This phase Ib study evaluated the safety and tolerability and clinical activity of lumretuzumab, administered in combination with pertuzumab and paclitaxel in patients with HER3-positive, HER2-low BC. Paclitaxel 80 mg/m^2^ every week (qw) was chosen as a chemotherapy backbone due to its widespread use in the therapy of MBC.

## Methods

### Study design

This phase Ib, open-label, dose-escalating study (ClinicalTrials.gov Identifier: NCT01918254) investigated the safety, pharmacokinetics (PK), pharmacodynamics (PD) and clinical activity of lumretuzumab in combination with pertuzumab and paclitaxel. The study was conducted in two phases: a dose escalation phase and an extension phase.

### Ethics

Local ethics committee approval was obtained and all patients provided written informed consent. The study was conducted in accordance with Good Clinical Practice guidelines and the Declaration of Helsinki in nine centers in Denmark, France, Germany and Spain.

### Patients

Patients had a histologically confirmed diagnosis of MBC and were either untreated (i.e. first line) or previously treated for MBC. Patients eligible for enrollment underwent a fresh (pretreatment) tumor biopsy that was used to assess the level of HER3 protein expression by IHC and central pathology review. HER3 expression was assessed by using a prototype IHC assay developed by Ventana Inc. and performed by Source Bioscience Ltd. Any discernible HER3 membrane staining in any neoplastic cell provided a minimum of 100 tumor cells were present in the biopsy specimen was considered positive for HER3 protein expression. At the same time, tumor biopsies had to show a low HER2 expression, i.e. IHC 1+/ISH- or IHC 2+/ISH- according to the ASCO HER2 Test 2007 Guidelines [14] and as assessed by parallel testing of protein and gene amplification at Source BioScience Ltd. (Nottingham, UK) using the Pathway HER2 IHC assay (Ventana Inc., USA) and the PathVysion HER2 FISH assay (Abbott Laboratories, USA).

### Study drug administration

Lumretuzumab and pertuzumab were administered every three weeks (q3w) and paclitaxel was administered every week (qw) as an IV infusion defining a treatment cycle of 21 days.

As shown previously, lumretuzumab PK was linear for doses of at least 400 mg IV, indicative of target-mediated drug disposition (TMDD) saturation, and PD activity was demonstrated for monotherapy when administered every two weeks (q2w) [10]. The TMDD model predicted that a lumretuzumab dose of ≥800 mg would be required to ensure adequate serum levels and target saturation for the entire dosing period when administered q3w [15]. Therefore, the dose of 1000 mg was considered as the starting dose for the combination treatment in Cohort 1. In Cohorts 2 and 3, the dose was reduced to 500 mg. For pertuzumab, the standard dose (840 mg loading dose [LD] followed by 420 mg at the following infusions) was used in Cohorts 1 and 2. In Cohort 3, pertuzumab doses of 420 mg for the first and following infusions was administered. Paclitaxel was given at a standard dose of 80 mg/m^2^ for all cohorts. No dose reductions were allowed for pertuzumab or lumretuzumab. In case of paclitaxel-related toxicities, the dose of paclitaxel could be reduced once to 60 mg/m^2^. Paclitaxel, pertuzumab and lumretuzumab administration could be delayed to assess or treat related adverse events (AEs) for up to 21 days.

Prophylactic antidiarrheal treatment with loperamide was introduced in Cohort 3 and consisted of: 4 mg prior to Cycle 1 followed by 2 mg every 4 h on Day 1 of Cycle 1, 2 mg every 4 h from Day 2 to 4 of Cycle 1, 1 mg every 6 to 8 h between Day 5 to 21 of Cycle 1, and titrated as needed for all subsequent cycles.

Patients continued treatment until disease progression, unacceptable toxicity or withdrawal of consent.

### Tumor response and safety assessments

Tumor response assessment using Response Evaluation Criteria in Solid Tumors (RECIST) version 1.1 [16] was conducted at screening and every 9 weeks thereafter.

Safety assessments included physical (ECOG performance status, vital signs) and laboratory examinations, electrocardiogram and echocardiogram. AEs were defined according to the Common Terminology Criteria for Adverse Events, version 4.03 (CTCAEv4.03).

### Definition of dose-limiting toxicity (DLT)

For Cohort 1, a DLT was defined as an AE occurring during the first cycle of treatment with lumretuzumab and considered study drug-related. For Cohorts 2 and 3, the DLT period was extended to two treatment cycles. AEs qualifying as DLTs included: grade 4 neutropenia (i.e. absolute neutrophil count [ANC] < 0.5 × 10^9^ cells/L for minimal duration of seven days); grade 3 and 4 febrile neutropenia; grade 4 thrombocytopenia; grade 3 thrombocytopenia associated with bleeding episodes; grade ≥ 3 non-hematological toxicity; failure to recover from any treatment-related toxicity grade ≥ 2 which results in a dose delay of >14 days of the next scheduled administration; grade 3 neuropathy that causes a dose delay of >14 days. IRRs, alopecia, grade 3 nausea and vomiting and diarrhea that respond to optimal management, grade 3 diarrhea lasting for ≤2 days with no fever or dehydration and laboratory values of ≥ grade 3 which were judged not clinically significant by the investigator were not considered DLTs.

### Pharmacokinetic assessments

PK evaluation was conducted for all patients on Day 1 to 20 of Cycle 1. PK parameters (area under the serum concentration–time curve [AUC], maximum-observed serum concentration [C_max_], half-life [t_1/2_], volume of distribution [V_d_] and clearance [CL]) for lumretuzumab and pertuzumab were computed by non-compartmental analysis (NCA; WinNonlin Version 6.4.0, Pharsight Corp.).

### Biomarker assessments

Fresh tumor biopsies were collected during screening. HER3 and HER2 protein expression was assessed using an IHC assay, scored semi-quantitatively and reported as an Immuno-reactive Score (IRS, range 0 to 3) as described previously [10].

Heregulin (HRG) mRNA expression, considered a potential predictive biomarker for lumretuzumab activity, was measured by quantitative real-time PCR (qRT-PCR) assay at Roche Molecular Systems (Pleasanton, USA) in screening formalin-fixed, paraffin-embedded (FFPE) tumor biopsies from a limited number of patients (*n* = 8).

Total RNA was isolated from FFPE tumor tissue sections using the Cobas® RNA isolation kit. Taqman probes were designed to detect HRG and respective reference genes simultaneously. All reagents were prepared at Roche Molecular Systems and qRT-PCR was performed using the Cobas® 4800 system. Calculation of the cycle-to-threshold (Ct) for each fluorescent channel was done using LC480 software and the relative log HRG expression was reported as ΔCt where ΔCt = Ct(Reference) – Ct(Target). Where feasible biopsies with less than 50% tumor content underwent macro-dissection guided by pathologist annotation of adjacent hematoxylin and eosin (H&E)-stained sections. No reference ranges were defined for HRG mRNA expression using the research grade assay.

Tumor DNA mutations were investigated in all patients where sufficient baseline FFPE material was available using the FoundationOne® version T7 genomic profiling assay (Foundation Medicine Inc., Cambridge, MA, USA) [17].

### Statistical considerations

All patients who received at least one dose of study medication were included in the safety and efficacy population. Descriptive statistics were used for demographics and safety, as well as for efficacy and biomarkers. In addition, in order to evaluate the potential relationship of predose expression of HER3 and HER2 with clinical response, a Fisher’s Exact Test for assessing the association between baseline value of the biomarker (IRS score – below or above median - or IHC score) and the presence or absence of response was used.

## Results

### Patients

Patient demographics and baseline characteristics are presented in Table 1. In the dose escalation phase, 2 patients were initially treated with 1000 mg of lumretuzumab in Cohort 1. DLTs occurred in both patients (diarrhea grade 3, hypokalemia grade 4 and hyponatremia grade 3 in Cycle 1 in one patient; and diarrhea grade 3 in Cycle 1 in the other patient). Subsequently, six patients were tested at a reduced dose of 500 mg of lumretuzumab in Cohort 2, and no DLTs were reported. Another 14 patients were enrolled into Cohort 2 as an extension phase. Due to the unfavorable safety profile with regard to diarrhea in Cohort 2, Cohort 3 was initiated. Based on the PK profile of lumretuzumab, we expected that further dose reductions would lead to the loss of linear PK and rapid clearance of lumretuzumab. Therefore, and because of the early onset of diarrhea, we omitted the LD of pertuzumab as the next step of dose modification and introduced a prophylactic anti-diarrheal medication for Cohort 3. No DLTs were reported in the first 6 patients and another seven patients were enrolled into Cohort 3.

**Table 1:** Baseline patient demographics and characteristics

Characteristic	Cohort 1 *N* = 2	Cohort 2 *N* = 20	Cohort 3 *N* = 13	Overall *N* = 35
Age, median (range), years	52.5 (42, 63)	62.0 (35, 75)	49.0 (33, 77)	60.0 (33, 77)
Sex, n (%)				
Male	0	0	1 (7.7)	1 (2.9)
Female	2 (100)	20 (100)	12 (92.3)	34 (97.1)
ECOG score, median (range)	0.5 (0, 1)	0 (0, 1)	0 (0, 1)	0 (0, 1)
ER-positivity of primary tumor, no. of patients (%)	1 (50.0)	16 (80.0)	12 (92.3)	29 (82.9)
ER-positivity of recurrent tumor, no. of patients (%)	1 (50.0)	11 (55.0)	9 (75.0)	21 (61.8)
Previous lines of chemotherapy for MBC, median (range)	0.5 (0, 1)	1 (0, 5)	0	0 (0, 5)
No. of cycles, median (range)				
Paclitaxel	2 (2, 2)	3.5 (1, 7)	6 (1, 10)	–
Pertuzumab	2 (2, 2)	5 (1, 23)	5 (1, 19)	–
Lumretuzumab	2 (2, 2)	5.5 (1, 23)	7 (1, 19)	–

*ER* estrogen receptor, *MBC* metastatic breast cancer, *n* number of patients

Overall, 23 patients (65.7%) discontinued the study due to progressive disease, eight patients (22.9%) were withdrawn due to an AE (considered related to study treatment in 7 patients [20.0%]), two patients (5.7%) refused further treatment, and two patients (5.7%) were withdrawn at the discretion of the investigator.

### Safety

A total of 657 AEs were reported in 35 patients (Table 2). The most frequent AEs, irrespective of relationship to study treatment, included diarrhea (35 patients [100%]), nausea (24 patients [68.6%]), hypokalemia (20 patients [57.1%]) and weight loss (18 patients [51.4%]). The most frequent ≥ grade 3 AEs included diarrhea (16 patients [45.7%]) and hypokalemia (14 patients [40.0%]). AEs leading to withdrawal from the study in 9 patients (25.7%) were: diarrhea (6 patients [17.1%]), left ventricular dysfunction, weight loss and decreased appetite (1 patient [2.9%] each).

**Table 2:** Summary of adverse events of any grade and of grade ≥ 3 adverse events irrespective of the relationship to study treatment

Adverse event	No. of patients having an adverse event (%)							
	Cohort 1 N = 2		Cohort 2 N = 20		Cohort 3 N = 13		All patients N = 35	
	All grades	Grade ≥ 3	All grades	Grade ≥ 3	All grades	Grade ≥ 3	All grades	Grade ≥ 3
Diarrhea	2 (100)	2 (100)	20 (100)	10 (50.0)	13 (100)	4 (30.8)	35 (100)	16 (45.7)
Nausea	1 (50.0)	0	16 (80.0)	0	7 (53.8)	0	24 (68.6)	0
Hypokalemia	2 (100)	1 (50.0)	13 (65.0)	11 (55.0)	5 (38.5)	2 (15.4)	20 (57.1)	14 (40.0)
Weight loss	1 (50.0)	0	13 (65.0)	4 (20.0)	4 (30.8)	0	18 (51.4)	4 (11.4)
Alopecia	0	0	8 (40.0)	0	9 (69.2)	0	17 (48.6)	0
Decreased appetite	1 (50.0)	0	12 (60.0)	1 (5.0)	2 (15.4)	1 (7.7)	15 (42.9)	2 (5.7)
Rash	0	0	9 (45.0)	1 (5.0)	4 (30.8)	0	13 (37.1)	1 (2.9)
Asthenia	2 (100)	1 (50.0)	7 (35.0)	1 (5.0)	3 (23.1)	1 (7.7)	12 (34.3)	3 (8.6)
Hypomagnesemia	1 (50.0)	1 (50.0)	8 (40.0)	0	3 (23.1)	0	12 (34.3)	1 (2.9)
Vomiting	0	0	6 (30.0)	0	6 (46.2)	0	12 (34.3)	0
Urinary tract infection	0	0	6 (30.0)	0	4 (30.8)	1 (7.7)	10 (28.6)	1 (2.9)
Mucosal inflammation	1 (50.0)	0	4 (20.0)	0	5 (38.5)	0	10 (28.6)	0
Dygeusia	0	0	7 (35.0)	0	3 (23.1)	0	10 (28.6)	0
Fatigue	0	0	4 (20.0)	0	5 (38.5)	0	9 (25.7)	0
Pyrexia	1 (50.0)	0	6 (30.0)	0	2 (15.4)	0	9 (25.7)	0
Nasopharyngitis	0	0	3 (15.0)	0	6 (46.2)	0	9 (25.7)	0
ALT increased	0	0	7 (35.0)	4 (20.0)	1 (7.7)	0	8 (22.9)	4 (11.4)
Infusion-related reaction	0	0	5 (25.0)	0	3 (23.1)	0	8 (22.9)	0
Neurotoxicity	0	0	4 (20.0)	3 (15.0)	3 (23.1)	1 (7.7)	7 (20.0)	4 (11.4)
AST increased	0	0	6 (30.0)	2 (10.0)	1 (7.7)	0	7 (20.0)	2 (5.7)
Abdominal pain	1 (50.0)	0	5 (25.0)	0	1 (7.7)	0	7 (20.0)	0
Anemia	1 (50.0)	0	3 (15.0)	0	3 (23.1)	0	7 (20.0)	0
Hypophosphatemia	1 (50.0)	1 (50.0)	2 (10.0)	1 (5.0)	3 (23.1)	3 (23.1)	6 (17.1)	5 (14.3)
Epistaxis	0	0	2 (10.0)	0	4 (30.8)	0	6 (17.1)	0
Headache	0	0	3 (15.0)	0	3 (23.1)	0	6 (17.1)	0
Polyneuropathy	0	0	5 (25.0)	0	1 (7.7)	0	6 (17.1)	0
Flatulence	0	0	3 (15.0)	0	3 (23.1)	0	6 (17.1)	0
Lymphopenia	0	0	1 (5.0)	0	3 (23.1)	2 (15.4)	4 (11.4)	2 (5.7)
Constipation	0	0	2 (10.0)	1 (5.0)	3 (23.1)	0	5 (14.3)	1 (2.9)
Edema peripheral	0	0	2 (10.0)	0	3 (23.1)	0	5 (14.3)	0
Hypocalcemia	1 (50.0)	0	2 (10.0)	0	2 (15.4)	0	5 (14.3)	0
Dry mouth	0	0	4 (20.0)	0	0	0	4 (11.4)	0
Stomatitis	0	0	2 (10.0)	0	2 (15.4)	0	4 (11.4)	0
Neuropathy peripheral	0	0	2 (10.0)	0	2 (15.4)	0	4 (11.4)	0
Acne	0	0	2 (10.0)	0	2 (15.4)	0	4 (11.4)	0
Erythema	0	0	3 (15.0)	0	1 (7.7)	0	4 (11.4)	0
Onycholysis	0	0	2 (10.0)	0	2 (15.4)	0	4 (11.4)	0
Pruritus	0	0	3 (15.0)	0	1 (7.7)	0	4 (11.4)	0
Back pain	0	0	2 (10.0)	0	2 (15.4)	0	4 (11.4)	0
Pain in extremity	0	0	3 (15.0)	0	1 (7.7)	0	4 (11.4)	0

Only adverse events reported by >10% of the patients overall are shown. Adverse events are ordered by decreasing frequency for all grade events in the overall population

N = number of patients

Diarrhea and hypokalemia were the most prominent AEs in this study. Both patients of Cohort 1 (100%) had ≥ grade 3 diarrhea. In Cohort 2, 10 patients (50.0%) had grade 1/2 diarrhea as highest grade and 10 patients (50.0%) had ≥ grade 3 diarrhea; and 2 patients (20.0%) had grade 1/2 hypokalemia and 11 patients (55.0%) had ≥ grade 3 hypokalemia, all occurring concomitantly with an episode of diarrhea. Reducing the dose of paclitaxel from 80 to 60 mg/m^2^ or interrupting the dose did not have an impact on the course of diarrhea and hypokalemia in Cohort 2. In Cohort 3, after implementation of loperamide prophylaxis and omission of the LD of pertuzumab, 9 patients (69.2%) had grade 1/2 diarrhea and 4 patients (30.8%) had ≥ grade 3 diarrhea; and 3 patients (23.1%) had grade 1/2 hypokalemia and 2 patients (15.4%) had ≥ grade 3 hypokalemia.

### Pharmacokinetic analysis

Lumretuzumab PK parameters at Cycle 1 are shown in Table 3. Lumretuzumab PK parameters in Cohort 1 and 2 were in the expected range for a 500 mg dose based on the first-in-human dose escalation study of lumretuzumab as monotherapy [10]. Data from this previous study indicated that lumretuzumab ≥400 mg was in the dose-linear range and target saturation would be ≥95% over the dosing interval. The similarities in PK parameters to those following lumretuzumab monotherapy indicate that there is no influence of pertuzumab on the PK of lumretuzumab. Only two patients received lumretuzumab at 1000 mg (Cohort 1).

**Table 3:** Serum PK parameters following the first administration

Cohort	Descriptive statistic	C_max_ (μg/mL)	AUC_last_ (day x μg/mL)	V_d_ (mL)	Total CL (mL/day)	t_1/2_ (day)
Lumretuzumab						
1	*N*	2	2	2	2	2
	Mean	448	3180	3370	253	10.1
	CV%	8.52	27	4.1	44.6	40.9
2	*N*	19	18	18	18	18
	Mean	188	1430	4040	275	11
	CV%	18.6	23.3	25.7	32.3	34.5
3	*N*	10	8	9	9	9
	Mean	161	1160	4330	295	10.9
	CV%	18.8	10.8	26.7	26.6	40.7
Pertuzumab						
1	*N*	2	1	ND	ND	ND
	Mean	155	2160	ND	ND	ND
	CV%	15.1	ND	ND	ND	ND
2	*N*	13	7	10	10	10
	Mean	256	2560	4920	229	15.6
	CV%	35	34.1	36.5	35.7	33.5
3	*N*	10	4	10	10	10
	Mean	157	1260	4440	240	13.5
	CV%	12.7	27.2	31.4	33.8	27.2

Abbreviations: *AUC*_*last*_ area under the concentration-time curve up to the last measurable concentration, *C*_*max*_ maximum-observed serum concentration, *CL* total clearance *ND* not determined, *t*_*1/2*_ half life, *V*_*d*_ volume of distribution

Pertuzumab PK parameters at Cycle 1 are shown in Table 3. For pertuzumab, following administration of a LD of 840 mg (Cohort 1 and 2), the pertuzumab PK was similar to that observed in patients with MBC [11]. Following administration of pertuzumab at 420 mg for Cycle 1 (Cohort 3) the exposure (C_max_ and AUC_last_) was approximately 50% of the exposure observed following a 840 mg LD (Cohorts 1 and 2), with clearance, volume of distribution and half-life remaining in the same range.

### Biomarker analysis

All patients enrolled had tumors that were HER3-positive based on IHC analysis at screening. Retrospective analysis of freshly stained FFPE tumor biopsy sections indicated that HER3 was highly expressed on the membranes of tumor cells (median [range] IRS: 2.21 [1.04 to 3], *n* = 35). HER2 expression at screening was scored according to published guidelines [18] for inclusion purposes, whilst an IRS score was calculated for further biomarker comparisons. Tumors of all patients enrolled were HER2-low as described above (HER2 IHC score [n]: 1+ [23]; 2+ [12]), with a median (range) IRS of 0.12 (0.0001 to 1.9, n = 35). Neither baseline HER2 nor HER3 expression was associated with clinical response (all *p*-values from Fisher’s exact tests were non-significant: p-value for HER2 IHC score was 1; for membranous HER2 IRS: 1; for membranous HER3 IRS: 0.72).

HRG mRNA expression was determined in a small cohort of patients and compared to expression in patients with squamous NSCLC treated with lumretuzumab and erlotinib in a previous study, where HRG was investigated as a potential predictive marker of lumretuzumab activity [19]. In the present study, HRG mRNA was lower for MBC patients than that observed in squamous NSCLC patients (median delta Ct [range]): MBC -3.35 [−6.78 to −1.4], *n* = 8; squamous NSCLC -0.72 [−5.12 to 1.64], *n* = 15). There was no apparent relationship with clinical response.

DNA sequencing data was available from predose FFPE tumor biopsies for 32 out of 35 patients (91.4%). Overall, 564 short variant mutations of known, likely or unknown function were found across 236 genes with a median read depth of 584 (range 90 to 2672). Germline mutations in genes directly linked to the HER pathway were retained whilst 268 other annotated germline mutations were censored in the data set leaving 296 mutations from 196 genes across 32 patients. PIK3CA and TP53 were the most commonly observed mutated genes (13 out of 32 patients [40.6%] and 11 out of 32 patients [34.4%], respectively) (Table 4). Common PIK3CA mutations were H1047R, E542K and E545A/K, each found in 3 different patients. There was no association of PIK3CA mutations with response. The most commonly mutated genes are listed in Table 4.

**Table 4:** Most prevalent gene mutations observed in predose formalin-fixed, paraffin-embedded tumor biopsies using the FoundationOne® genomic profiling assay

Gene	Prevalence (n)	Total mutations	Mutations (n)
PIK3CA	40.6% (13)	15	H1047R (4), E542K (3), E545K (3), E545A (1), E365Q (1), P124L (1), N1068 fs*3+ (1), G106-E109del (1)
TP53	34.4% (11)	11	Q52H (1), V157E (1), V173 M (1), R175M (1), L194R (1), Y220C (1), S241F (1), R248Q (1), R248W (1), R280T (1), L194R (1)
GATA3	28.1% (9)	12	P191H (1), H237fs*29 (1), H282_Y283 > QHY (1), M357I (2), S402 fs*45+ (1), P409fs*38+ (1), S427 fs*20+ (1), T441 fs*6+ (1), M443 fs*4+ (1), A70D (1), P42L (1)
MLL3	18.8% (6)	9	R2296H (1), K2797 fs*26 (1), N2990 fs*4 (1), E4049* (1), R4595G (1), R4139* (1), E1748K (1), E1746* (1), C438Y (1)
ESR1	18.8% (6)	7	D538G (3), S118P (1), T496 N (1), L536P (1), L549P (1)
BRCA2	15.6% (5)	6	R1190W (1), I1859fs*3 (1), R2027K (1), K2404 fs*7 (1), R2502H (1), K3326* (1)
LRP1B	15.6% (5)	5	A960V (1), E2998K (1), R2443H (1), R2777* (1), H112Y (1), S3586 T (1)
SPEN	12.5% (4)	9	R187W (1), T838R (1), I1159fs*28 (1), N2072 fs*51 (1), E2176* (1), T3104 M (1), A3169V (1), E2260K (1), S2120F (1)
ARID1A	12.5% (4)	6	S1138 fs*55 (1), G1711A (1), V1834A (1), D1850fs*33 (1), Q2176fs*48 (1), Splice site 3406 + 1G > T (1)
JAK1	12.5% (4)	6	G319 W (1), Q834* (1), R839Q (1), K860 fs*16 (1), E1051* (1), splice site 2404-1G > A (1)

n = number of patients with mutations

Single nucleotide polymorphism gene mutations annotated as: gene, amino acid change. Tabulated mutations include those of known status (short-variants that are recurrent somatic; copy-number alterations involving genes that are recurrently amplified/deleted; rearrangements involving known fusion partners, or other known functional events), likely status (short-variants that disrupt tumor suppressor genes or are in known hotspot regions; rearrangements that disrupt tumor suppressor genes or other likely functional events) and unknown status (variants with unknown somatic/functional status). Additionally germline mutations in genes linked to the ErbB3 pathway are included in the overall analysis (n = 20 mutations). Full listing of mutations is provided in Supplementary Table 2

**Table 5:** Tumor response to treatment (RECIST)

	Number of patients (%) with respective assessment			
	Cohort 1 N = 2	Cohort 2 N = 20	Cohort 2a N = 9	Cohort 3 N = 13
Complete response	0	1 (5.0)	1 (11.1)	0
Partial response	0	5 (25.0)	4 (44.4)	5 (38.5)
Stable disease	2 (100)	9 (45.0)	2 (22.2)	5 (38.5)
Progressive disease	0	4 (20.0)	1 (11.1)	2 (15.4)
Lost to follow up	0	1 (5.0)	1 (11.1)	1 (7.7)
Objective response rate	0	6 (30.0)	5 (55.5)	5 (38.5)
Disease control rate	2 (100)	15 (75.0)	7 (77.7)	10 (76.9)

N = number of patients

Cohort 2a includes first-line patients of Cohort 2 only

HER3 and HER2 mutations were found in 1 and 2 out of 32 patients (3.1% and 6.3%, respectively). HER3 V104 M and E928G mutations were found in Patient 1374 (best RECIST response of stable disease [SD]) which were heterozygous with allele frequencies of 39% and 29%, respectively, potentially conferring a degree of ligand-independent activation and increased constitutive phosphotransferase activity [20].

### Antitumor activity

**Fig. 1:**
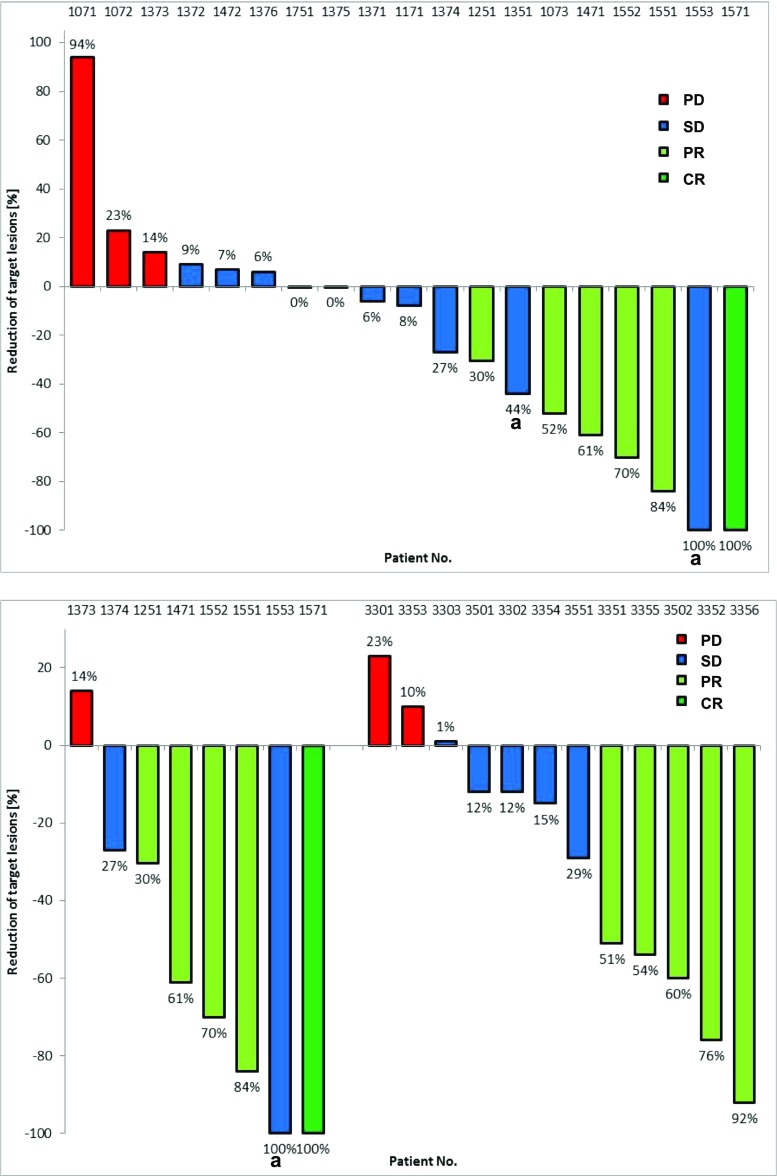
Best percent change from baseline in sum of target lesions and best confirmed response according to RECIST. **a** Cohort 2 patients and **b** first-line patients of Cohort 2 (left) and patients of Cohort 3 (right). ^a^ Unconfirmed partial response. Two patients are not shown who were discontinued prior to the first on-treatment tumor assessment

Clinical activity outcomes are indicated in Table 5 and best percentage change from baseline in sum of target lesions is shown in Fig. 1. For the 20 patients enrolled in Cohort 2, the objective response rate (ORR i.e. % partial response [PR] + complete response [CR]) was 30.0%, the disease control rate (DCR, i.e. % SD + PR + CR) was 75.0% and median PFS was 4.2 months. In those patients of Cohort 2 who had not received chemotherapy for MBC (first-line patients) (*n* = 9) the ORR was 55.5%, DCR was 77.7% and median PFS was 6.2 months. In the 13 patients of Cohort 3, all of which were first-line patients, the ORR was 38.5%, the DCR was 76.9% and the median PFS was 8.2 months.

## Discussion

In this phase Ib study the combination of lumretuzumab plus pertuzumab added to a chemotherapy backbone of paclitaxel was evaluated in patients with HER3-positive, HER2-low expressing MBC.

The initial overall response rate of 55.5% in patients of Cohort 2 who had not been previously treated with chemotherapy for MBC was encouraging and compared favorably to previous reports of the ORR with single agent weekly paclitaxel treatment ranging from 21% to 33% [21, 22]. Based on these data, Cohort 3 (with a reduced pertuzumab LD and prophylactic loperamide treatment to mitigate diarrhea) was initiated and included first-line MBC patients only to decrease heterogeneity of the population in comparison to Cohorts 1 and 2. With an ORR of 38.5% in Cohort 3, the response rate of first-line patients in Cohort 2 could not be confirmed. PFS was in the expected range for paclitaxel monotherapy [22].

Previous reports have demonstrated that response to HER3-targeting therapy may be associated with the increased gene expression of HER2 and HER3 [23] or HRG [24]. Similarly, in HR-positive, HER2-negative MBC patients, increased PFS was observed for patients with tumors expressing higher levels of HRG mRNA when treated with a HER3-targeting monoclonal antibody plus anti-hormonal therapy as compared to anti-hormonal therapy alone [25]. In the present study, however, tumor HER2 and HER3 protein expression were not associated with clinical response. HRG mRNA expression was generally too low to enable an association to clinical activity. Mutational analyses revealed that PI3K and TP53 mutations were amongst the most frequently mutated genes with a prevalence in the expected range for MBC [26]. Again no clear association with response to therapy could be shown. Overall our data suggest that HER2-low expressing tumors may not depend on either HER2/HER2, HER2/HER3, EGFR/HER2, or EGFR/HER3 signaling dimers and that the escape mechanisms to lumretuzumab + pertuzumab therapy may be multifaceted or simply undetectable in our cohort of MBC patients.

In this study, diarrhea accompanied by hypokalemia was the major toxicity across all dose cohorts. In Cohort 1, two out of two patients experienced grade 3/4 diarrhea and hypokalemia that were both considered dose-limiting. In a first step, the lumretuzumab dose was reduced from 1000 mg to 500 mg and no DLTs were reported in Cohort 2. Nevertheless, the incidence of diarrhea and hypokalemia was high (≥ grade 3 diarrhea: 50.0%; ≥ grade 3 hypokalemia: 55.0%). As the onset of diarrhea was within the 1st cycle for the vast majority of patients, we omitted the LD of pertuzumab as a next step in order to significantly decrease the exposure in the first cycle. Furthermore, we introduced a prophylactic loperamide treatment regimen and an intensified blood electrolytes monitoring. Overall, a reduction in the incidence of ≥ grade 3 diarrhea to 30.8% and of ≥ grade 3 hypokalemia to 15.4% in Cohort 3 was reported. Notwithstanding that prophylactic loperamide intake decreased the severity of diarrhea, chronic diarrhea remained the major toxicity. Diarrhea observed in this study exceeded the incidence of previously described reports of HER3-targeting drugs in combination with trastuzumab [27–30], or for pertuzumab therapy alone or in combination with trastuzumab [5, 31–33]. Diarrhea observed in the present study was likely due to complete inhibition of HER family dimers in the intestinal epithelium. EGFR, HER2 and HER3 are expressed on intestinal epithelial cell membranes and act in concert to negatively regulate chloride secretion via the PI3K and PKC pathways [34, 35]. The physiological mechanisms causing diarrhea were further investigated by a dedicated set of in vitro studies in human colon cell lines and tissue explants using lumretuzumab and pertuzumab. These experiments confirmed disinhibition of chloride channel activity in colonocytes by HER signaling inhibition as the mechanism underlying the secretory diarrhea reported in patients [36].

## Conclusions

Despite multiple mitigation efforts by dose modifications of study drugs and prophylactic loperamide treatment, chronic diarrhea remained the major side effect. In addition, the promising initial antitumor activity could not be confirmed, limiting the options to improve the therapeutic index. The therapeutic window for the combination of lumretuzumab with pertuzumab and paclitaxel turned out to be too narrow to warrant further development in HER3-positive, HER2-low MBC. The strategy to combine HER3-targeting agents with EGFR-targeting agents has been tested in several clinical trials over the recent years and has yet failed to provide a clinically meaningful proof of concept [37–39]. In the light of the present study in HER3-positive, HER2-low MBC it is debatable whether combination of HER3-targeting agents with HER family inhibitors, particularly in tumors lacking a single molecular driver, is worth pursuing.

## Electronic supplementary material

ESM 1 (DOC 133 kb)

ESM 2 (XLSX 63 kb)
