# Supplementary material for: Phase Ib study evaluating safety and clinical activity of the anti-HER3 antibody lumretuzumab combined with the anti-HER2 antibody pertuzumab and paclitaxel in HER3-positive, HER2-low metastatic breast cancer
Source: Invest New Drugs. 2018 Jan 19;36(5):848–59. doi: 10.1007/s10637-018-0562-4 (PMC6153514; doi:10.1007/s10637-018-0562-4)
Supplement: Supplementary file 1 — (DOC 133 kb) [file 10637_2018_562_MOESM1_ESM.doc]

Supplementary Table 1 List of genes in Foundation One T7 panel

| **Gene** | ABL1, ABL2, ACVR1B, AKT1, AKT2, AKT3, ALK, ALOX12B, AMER1 (FAM123B), APC, APCDD1, AR, ARAF, ARFRP1, ARID1A, ARID1B, ARID2, ASXL1, ATM, ATR, ATRX, AURKA, AURKB, AXIN1, AXL, BACH1, BAP1, BARD1, BCL2, BCL2A1, BCL2L1, BCL2L2, BCL6, BCOR, BCORL1, BLM, BMPR1A, BRAF, BRCA1, BRCA2, BRD4, BRIP1, BTG1, BTK, C11orf30 (EMSY), CARD11, CASP8, CBFB, CBL, CCND1, CCND2, CCND3, CCNE1, CD274, CD79A, CD79B, CDC73, CDH1, CDH2, CDH20, CDH5, CDK12, CDK4, CDK6, CDK8, CDKN1A, CDKN1B, CDKN2A, CDKN2B, CDKN2C, CEBPA, CHD2, CHD4, CHEK1, CHEK2, CHUK, CIC, CRBN, CREBBP, CRKL, CRLF2, CSF1R, CTCF, CTNNA1, CTNNB1, CUL3, CUL4A, CUL4B, CYLD, CYP17A1, DAXX, DDR1, DDR2, DICER1, DIS3, DNMT3A, DOT1L, EGFR, EP300, EPHA3, EPHA5, EPHA6, EPHA7, EPHB1, EPHB4, EPHB6, ERBB2, ERBB3, ERBB4, ERCC4, ERG, ERRFI1, ESR1, EZH2, FAM175A, FAM46C, FANCA, FANCC, FANCD2, FANCE, FANCF, FANCG, FANCI, FANCL, FANCM, FAS, FAT1, FAT3, FBXW7, FGF10, FGF12, FGF14, FGF19, FGF23, FGF3, FGF4, FGF6, FGF7, FGFR1, FGFR2, FGFR3, FGFR4, FH, FLCN, FLT1, FLT3, FLT4, FOXL2, FOXP1, FRS2, FUBP1, GABRA6, GALNT12, GATA1, GATA2, GATA3, GATA4, GATA6, GEN1, GID4 (C17orf39), GLI1, GNA11, GNA13, GNAQ, GNAS, GPR124, GREM1, GRIN2A, GRM3, GSK3B, H3F3A, HGF, HLA-A, HLA-B, HLA-C, HNF1A, HOXB13, HRAS, HSD3B1, HSP90AA1, IDH1, IDH2, IGF1, IGF1R, IGF2, IGF2R, IKBKE, IKZF1, IL7R, INHBA, INPP4B, INSR, IRF2, IRF4, IRS2, JAK1, JAK2, JAK3, JUN, KAT6A (MYST3), KDM5A, KDM5C, KDM6A, KDR, KEAP1, KEL, KIT, KLHL6, KMT2A (MLL), KMT2C (MLL3), KMT2D (MLL2), KRAS, LMO1, LRP1B, LRP6, LTK, LYN, LZTR1, MAGI2, MAP2K1, MAP2K2, MAP2K4, MAP3K1, MAP3K13, MCL1, MDM2, MDM4, MED12, MEF2B, MEN1, MERTK, MET, MITF, MKNK1, MKNK2, MLH1, MPL, MRE11A, MSH2, MSH6, MST1R, MTOR, MUTYH, MYC, MYCL (MYCL1), MYCN, MYD88, NBN, NCOR1, NF1, NF2, NFE2L2, NFKBIA, NKX2-1, NOTCH1, NOTCH2, NOTCH3, NOTCH4, NPM1, NRAS, NSD1, NTRK1, NTRK2, NTRK3, NUDT1, NUP93, PAK3, PAK7, PALB2, PARK2, PARP1, PARP2, PARP3, PARP4, PAX5, PBRM1, PDCD1LG2, PDGFRA, PDGFRB, PDK1, PHLPP2, PIK3C2B, PIK3C2G, PIK3C3, PIK3CA, PIK3CB, PIK3CG, PIK3R1, PIK3R2, PLCG2, PMS2, PNRC1, POLD1, POLE, PPARG, PPP2R1A, PRDM1, PREX2, PRKAR1A, PRKCI, PRKDC, PRSS1, PRSS8, PTCH1, PTCH2, PTEN, PTPN11, PTPRD, QKI, RAC1, RAD50, RAD51, RAD51B (RAD51L1), RAD51C, RAD51D (RAD51L3), RAD52, RAD54L, RAF1, RANBP2, RARA, RB1, RBM10, REL, RET, RICTOR, RNF43, ROS1, RPA1, RPTOR, RUNX1, RUNX1T1, SDHA, SDHB, SDHC, SDHD, SETD2, SF3B1, SH2B3, SLIT2, SMAD2, SMAD3, SMAD4, SMARCA4, SMARCB1, SMARCD1, SMO, SNCAIP, SOCS1, SOX10, SOX2, SOX9, SPEN, SPOP, SPTA1, SRC, STAG2, STAT3, STAT4, STK11, SUFU, SYK, TAF1, TBX3, TEK, TERC, TERT (promoter only), TET2, TGFBR2, TIPARP, TNF, TNFAIP3, TNFRSF14, TNKS, TNKS2, TOP1, TOP2A, TP53. TP53BP1, TRRAP, TSC1, TSC2, TSHR, TYRO3, U2AF1, VEGFA, VHL, WISP3, WT1, XPO1, XRCC2, XRCC3, ZBTB2, ZNF217, ZNF703, ZNRF3 |
| --- | --- |
| **Gene rearrangement** | ALK, BCL2, BCR, BRAF, BRCA1, BRCA2, BRD4, EGFR, ETV1, ETV4, ETV5, ETV6, EWSR1, FGFR1, FGFR2, FGFR3, KIT, KMT2A (MLL), MSH2, MYB, MYC, NOTCH2, NTRK1, NTRK2, PDGFRA, RAF1, RARA, RET, ROS1, RSPO2, TMPRSS2 |

Supplementary Table 2 Gene mutations observed in predose formalin-fixed, paraffin-embedded tumor biopsies identified using the FoundationOne® genomic profiling assay
